# Deep Learning-Based Approach for Atrial Fibrillation Detection

**DOI:** 10.1007/978-3-030-51517-1_9

**Published:** 2020-05-31

**Authors:** Lazhar Khriji, Marwa Fradi, Mohsen Machhout, Abdulnasir Hossen

**Affiliations:** 8 Digital Research Centre of Sfax, Sfax, Tunisia; 9 Institut Mines-Télécom, CNRS, Paris, France; 10 Université de Sherbrooke, Sherbrooke, QC Canada; 11 Digital Research Centre of Sfax, Sfax, Tunisia; 12 University of Sfax, Sfax, Tunisia; 13 College of Engineering, Sultan Qaboos University, Muscat, Oman; 14 Faculty of Sciences of Monastir, Monastir University, Monastir, Tunisia

**Keywords:** ECG-classification, AF detection, Confusion matrix, ROC, ANN, Histogram error

## Abstract

Atrial Fibrillation (AF) is a health-threatening condition, which is a violation of the heart rhythm that can lead to heart-related complications. Remarkable interest has been given to ECG signals analysis for AF detection in an early stage. In this context, we propose an artificial neural network ANN application to classify ECG signals into three classes, the first presents Normal Sinus Rhythm NSR, the second depicts abnormal signal with Atrial Fibrillation (AF) and the third shows noisy ECG signals. Accordingly, we achieve 93.1% accuracy classification results, 95.1% of sensitivity, 90.5% of specificity and 98%. Furthermore, we yield a value of zero error and a low value of cross entropy, which prove the robustness of the proposed ANN model architecture. Thus, we outperform the state of the art by achieving high accuracy classification without pre-processing step and without high level of feature extraction, and then we enable clinicians to determine automatically the class of each patient ECG signal.

## Introduction

ECG signals classification is a crucial step to determine given the importance to assign to each patient its ECG class. Indeed, heart diseases have known a big spread in the last recent years. Such as arrhythmia cardiac problems like Atrial Fibrillation (AFIB). The prevalence of arterial fibrillation (AF) is increasing during the last few years and presenting the most common health problem in many countries [1]. AF presents a very critical health issue, which affects the quality of life of persons and leading to many risks such as cardiac stroke. An analysis of AF is based on a clinical evaluation and requires electrocardiogram (ECG) documentation during the arrhythmia. During the last few years, deep learning (DL) revolutionized the medical area as the deep neural networks presented the state of the art results in many applications such as computer vision, image processing, robotics, medical imaging, etc. The high performance obtained by the deep neural network is based on the use of powerful graphic processing units (GPUs) which allowed these implementations to outperform the classic ones. Because of the outstanding development in DL, its application in medical field (using biomedical signals) is of huge interest. Accordingly, many works were developed using DL models for ECG classification. In this context, our work in this paper presents a new contribution using artificial neural network (ANN) architecture, to classify MIT-BIH dataset signal into normal and AFIB ECG signals.

The rest of this work is divided into 4 sections. Section 2 summarizes the state of the art. Section 3 presents the propounded neural network model and experimental results. Discussions and conclusion are depicted in Sects. 4 and 5, respectively.

## State of the Art

In [2], Rahal et al. proposed a new approach for active classification of electrocardiogram ECG signals based on deep neural networks (DNN). Electrocardiogram ECG classification plays an essential role in clinical diagnosis of cardiac insufficiency. Zubair et al. in [3] proposed an ECG beat classification system based on convolutional neural network (CNN). This model is divided into two main parts, one for features extraction and the second for classification. Electrocardiogram ECG interpretation plays an important role in clinical ECG workflow. Rajpurkar et al. [4] developed a new method based on deep convolutional neural network to classify ECG signals belonging to fourteen different classes. Acharya et al. [5] did another study where they designed a novel deep CNN for ECG signals classification. Another work was proposed in [6] based on deep belief Net used for classifying heartbeats into four classes. A new method is presented in [7], which presents a new deep learning approach used for detecting atrial fibrillation in real time. In this work, authors used an end-to-end neural network combining a convolutional with a recurrent neural network (CNN, RNN) in order to extract high-level features from the input signals.

**Fig. 1.**
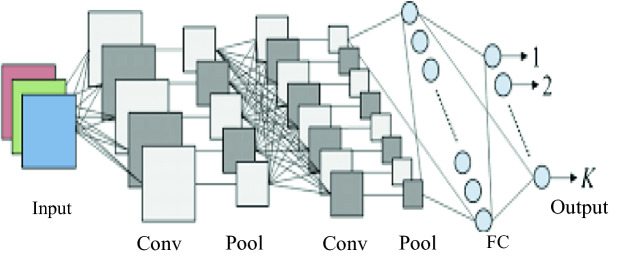
A CNN classical architecture

This hybrid model was trained and tested under three different datasets containing a total number of 85 classes. This model presents a particular performance by its ability of analyzing 24 h of ECG recordings in less than one second. This algorithm was tested on the three datasets in order to test its robustness and achieved the following results: 98.96% of specificity and 86.4% for sensitivity. Figure 1 presents a classic architecture of a Convolutional neural network (CNN).

There are many different approaches to the task of arrhythmia classification of ECG signals in terms of which method is used, which arrhythmias are classified, which data set is used, which features are extracted and whether individual beats or longer intervals are classified. For example, Rajpurkar et al. [4] uses a deep convolutional neural network trained on 30-s intervals of raw ECG signal data to classify 14 different classes, including normal sinus rhythm, noise, atrial fibrillation and atrial flutter.

**Fig. 2.**
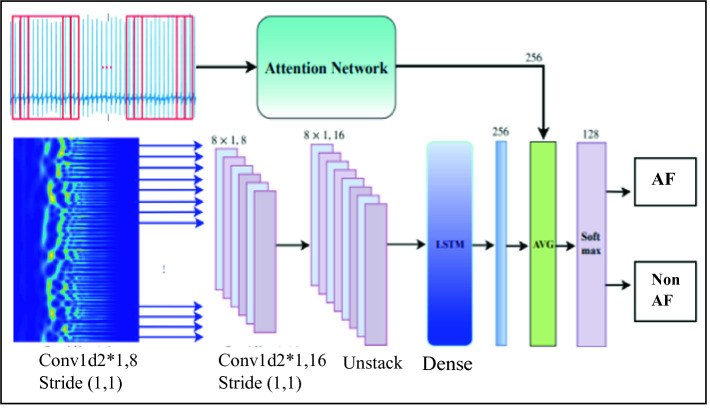
ECG-Net architecture

Atrial fibrillation presents a very complex input data for a neural network. Deep neural networks have shown a big performance in learning non-linear input data. As deep neural network is able to learn complex pattern presenting AF in ECG signal, these techniques can widely help researchers on finding parts that are more important on the ECG to focus on during the training set. Indeed, using a CNN results accuracy overcome 95% [8, 10]. Accordingly, in [13], authors introduce a 2-channels neural network in order to address the problem of AF presence in the ECG signals. This new neural network is named “ECGNet”. By using this model, authors achieved very encouraging results coming up to 99.4% as detection accuracy in MIT-BIH atrial fibrillation dataset with 5-s ECG segments. Figure 2 presents the architecture of the proposed ECGNet neural network. This DL technique has shown its ability to detect FA in a short time process. In addition, the Attention Network has achieved 99.8 of accuracy.

## Method and Results

### Method

#### Dataset

The MIT-BIH dataset is known by its popularity as it has served for a long time as an interesting reference to be useful for ECG signals classification and diagnosis detection. In this context, we use three types of records from MIT-BIT dataset such as 100 samples of Normal sinus rhythm NSR, 40 samples of Atrial Fibrillation ATFIB and finally 60 samples of Noisy ECG signals. Each sample constitutes of a matrix with a size of 3600 * 1, reaching a total of 202 * 3600 for the input ECG data. Records as depicted in Table 1 recognize each type of signals. For each record, an atrial fibrillation should be classified similarly as a specialist would, the annotated parameters have been labelled by specialist for a long time.

**Table 1.** MIT-BIH dataset

Records	Samples	Matrix-size of samples	Annotations
100,101,105,109,112,113,114,115,116,117	100	3600 * 1	NSR
201, 202, 203, 210, 219	42	3600 * 1	AFIB
205, 223, 207	60	3600 * 1	Noisy-ECG

**Fig. 3.**
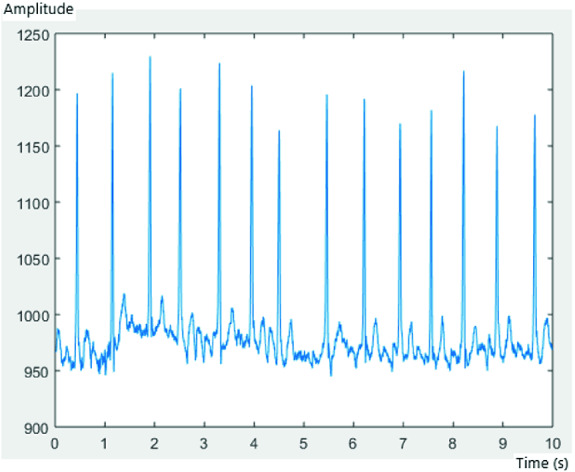
AFIB ECG of record 201

**Fig. 4.**
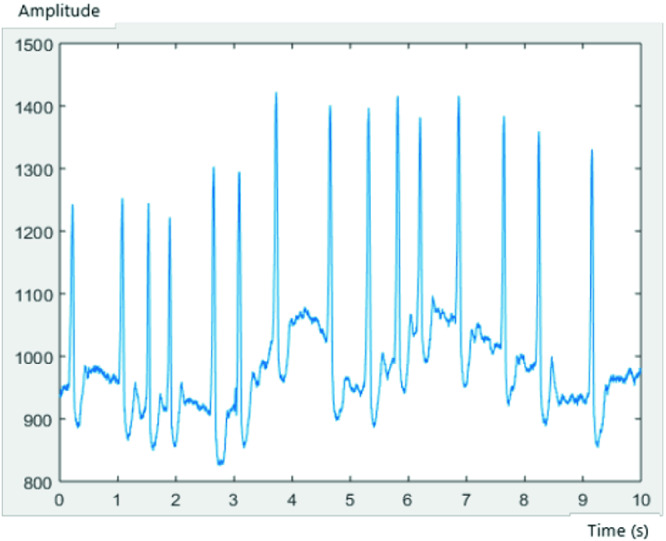
AFIB ECG of record 203

**Fig. 5.**
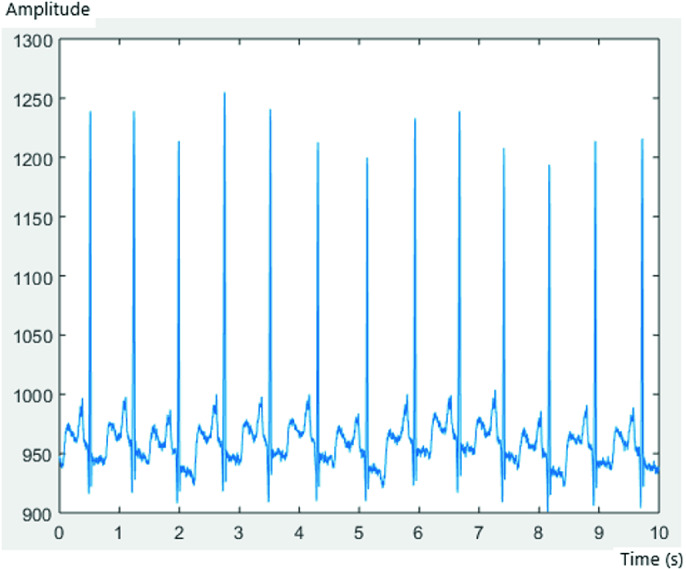
SNR ECG of record 100

Each record consisted of 3600 samples, with a frequency of sampling of 1/360 s. Figures 3, 4 and 5 represent an ECG signal of record 201, 203 and 100, respectively, with 3600 samples for each record.

#### Artificial Neural Network (ANN)

##### The Propounded ANN Architecture

**Fig. 6.**
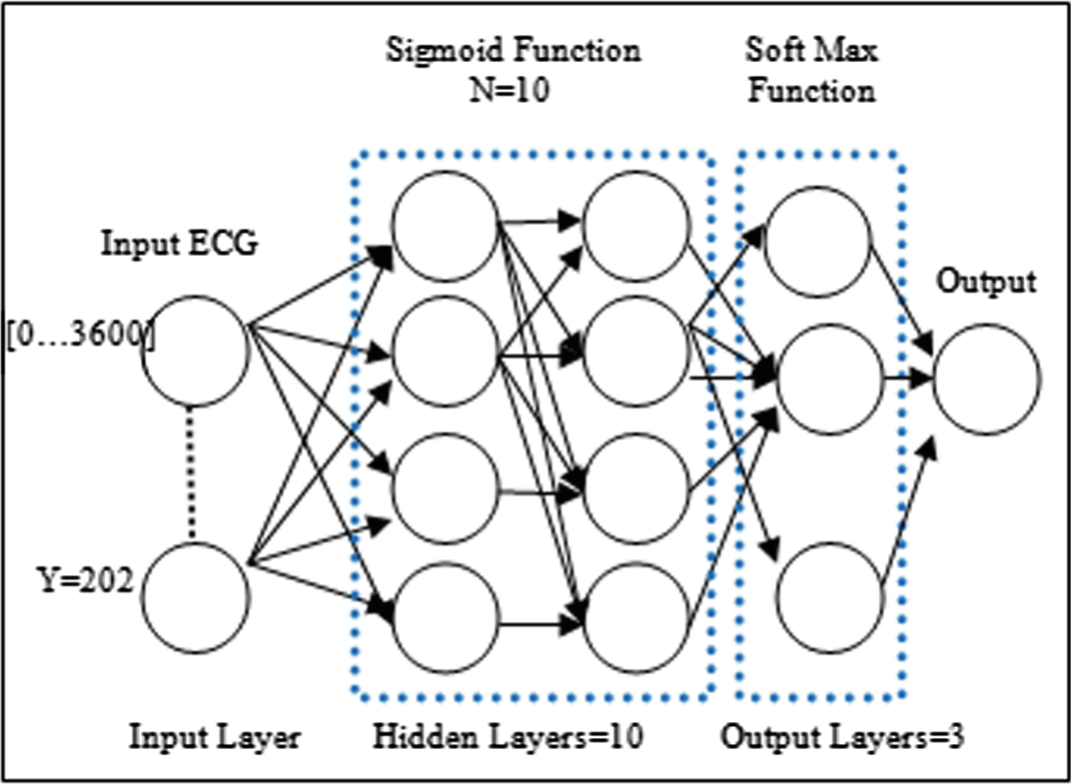
Synoptic flow of the proposed ANN architecture

**Fig. 7.**
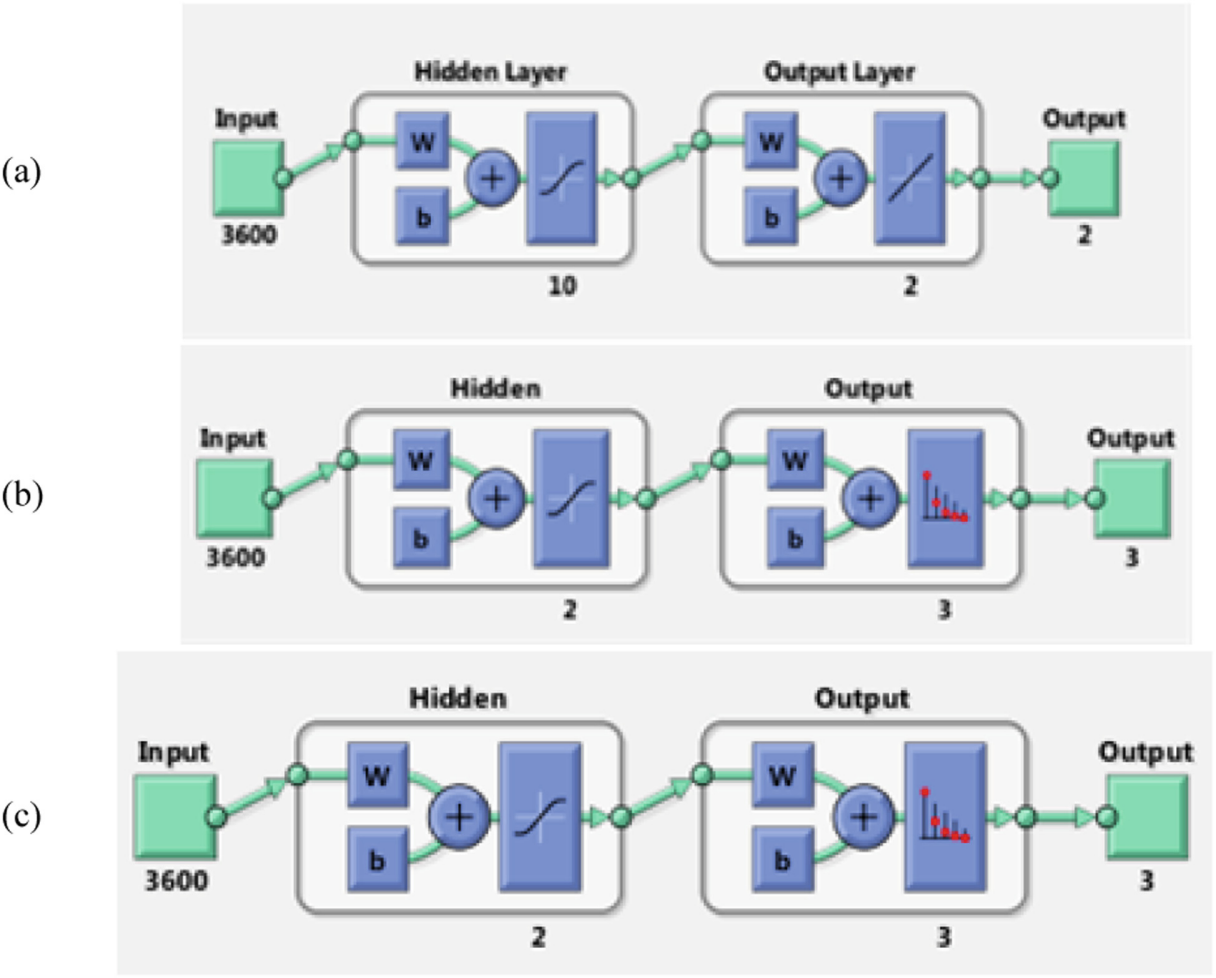
ANN trained architecture: (a) ANN with HL = 10, (b) ANN with HL = 2, (c) ANN with HL = 20.

The idea of creating an artificial neural network architecture was inspired by the biological neural system [12]. Indeed, our proposed artificial neural network consists on the input layer, which contains 202 samples of ECG signals, each sample consists of a matrix sized 3600 * 1; the input dataset is a 202 samples of vectors, constituting a matrix of 3600 * 202 of ECG signals. Then, a sigmoid activation function is applied, creating a respectful numbers of parameters, which present the feature maps of the ECG signals, passing through 10 hidden layer, and 10 neurons per layer, having 100 parameters, with a sigmoid activation function as depicted by the Eq. (1). Then a softmax function, presented by the Eq. (2), is applied to classify signals into three classes the first present an AFIB signal however, the second presents a SNR signal and the third depicts noisy ECG-signals. The suggested ANN architecture is depicted in Fig. 6 and Fig. 7 (a, b and c). The latter shows the used architecture when the number of Hidden Layers (HL) is 10, 2, and 20, respectively.

1 $$ y = \frac{1}{{1 + e^{ - x} }} $$

2 $$ Soft\hbox{max} (x_{i} ) = \frac{{e^{{x_{i} }} }}{{\sum\limits_{j} {e^{{x_{j} }} } }} $$

##### Training Data Parameters

Training parameters have a crucial role to obtain excellent accuracy. For this fact, the number of parameters should be done with the exact precision to get high accuracy, sensitivity and specificity results and to yield the best values. In fact, For ANN1, we use 10 hidden layer with 10 neurons for each layer. Thus, we obtain a number of 100 parameters, presenting the feature maps extracted from ECG signals. However, for ANN2 and ANN3, the number of hidden layers is 2 and 20 respectively. Thus, accuracy results will be discussed in the next section. Then, more the number of iterations is increasing; more classification results are going higher. Table 2 depicts all used parameters for the ECG classification process.

**Table 2.** Training data parameters

Training parameters	ANN1	ANN2	ANN3
Iterations	1000	1000	**1000**
Activation function	Sigmoid	Sigmoid	Sigmoid
Classifier function	Soft-max	Soft-max	Soft-max
Hidden layers	**10**	**2**	**20**
Parameters numbers	100	20	200
Train-samples	142	142	142
Validation-samples	30	30	30
Test-images	30	30	30
Error-rate	0.001	0.001	0.001
Batch	3600 samples	3600 samples	3600 samples

### ECG Classification Results

The propounded artificial neural network is presented with the confusion matrix, the histogram error, and the curve ROC and training performance results.

#### Confusion Matrix

**Fig. 8.**
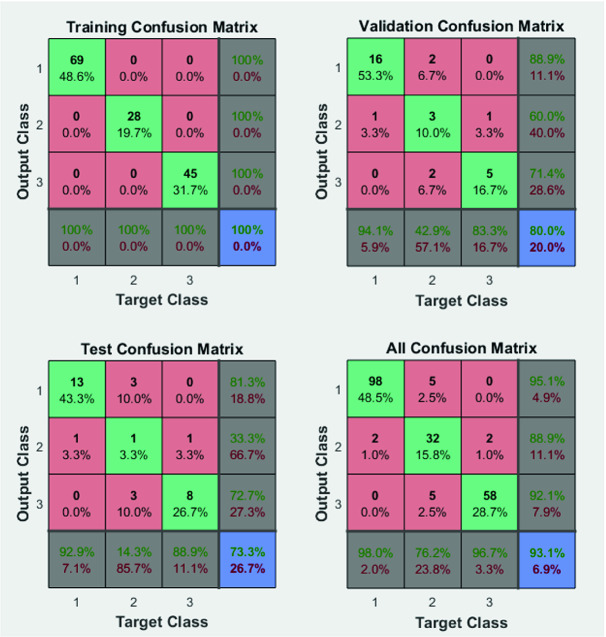
Confusion Matrix: training, validation and testing results with ANN1

A confusion matrix is a crucial method to determine the performance classification of a system since it divides the results into four classes such as True Positive (TP), True Negative (TN), False Positive (FP) and False Negative (FN). Accordingly, in Fig. 8, FP results are shown in the confusion matrix in the last row and the FN are depicted in the last column. It summarizes the prediction results on a classification issue. Therefore, it answers the problem of determination of the class of each signal. Indeed, it is depicted by a size of n × n associated with a classifier showing the predicted and actual classification, where n is the number of different classes. Table 1 shows a confusion matrix for n = 3. It gives us a sight not only into the errors being made by a classifier but more importantly the types of errors that are being made. The classification accuracy alone can be misleading where we have an unequal number of observations in each class or in case of having more than two classes in the dataset. Calculating a confusion matrix gives a better idea of what a classification model is getting right and what types of errors it is making. Indeed, performance results are calculated by the following equations presenting the sensitivity, the specificity and the accuracy.

3 $$ Sensitivity \, \left( {True \, positive \, rate} \right) \, = \frac{TP}{TP + FN} $$

4 $$ Specificity \, \left( {False \, positive \, rate} \right) \, = \frac{TN}{TN + FP} $$

5 $$ Accuracy \, \left( {percent \, of \, all \, samples \, correctly \, classified} \right) \, = \frac{TN + TP}{TN + TP + FN + FP} $$

The prediction results (Fig. 8) show an accuracy of 100% for the training process, 80% for the validation process and 73.3% for the testing process. These results presented a high precision of classification, which proves the robustness of the used artificial neural network architecture. Indeed, there are no misclassified signals in the training confusion matrix. However, in the validation confusion matrix in all confusion matrix a value of 0% for misclassified signal is achieved. The green colour in the confusion matrix represents the true positive classified ECG signals; however, the red colour depicts the misclassified signals. The first class presents the atrial fibrillation ECG signal, the second-class presents the SNR classified ECG signals. We used 142 records in the training process, where 69 records are classified as SNR signals, 28 records are classified as AFIB signals and 45 records are classified as noisy ECG signals, yielding a value of 100% of sensitivity and specificity. Moreover, for the validation stage, we used 30 records where 24 records are well-classified achieving accuracy value of 80%. Accordingly, test process achieved also a value of 73.3, where 22 records with 3600 samples for each record are well assigned to the right class, and 8 records are misclassified (3 records for the first class, 2 records for the second class and 3 records for the third class). These results are achieved due to the proposed architecture with 10 hidden layers, 10 neurons for each layer, with a sigmoid activation function and finally Softmax classifier proves its ability not seen before by the system. However, using a small number of hidden layers, the accuracy classification is with 82.6% and 87% using a high number of layers. Thus, the choice of a precision number of hidden layers for getting precise number of parameters shows its worth in the ECG-classification process.

#### Histogram Error

**Fig. 9.**
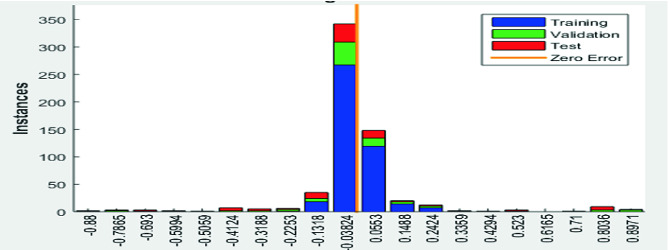
Histogram error results where Errors = Targets−Outputs

The histogram error determines the rate of the existent error in classifying the signals used for training, for validation and for testing. The precision shows whether the classification is well done or not (i.e. with errors). Indeed, in our ECG signals classification results yield roughly 0.07% errors as depicted in Fig. 9 where the data fitting errors are presented within a reasonably good range close to zero.

#### Receiver-Operating Curve (ROC)

**Fig. 10.**
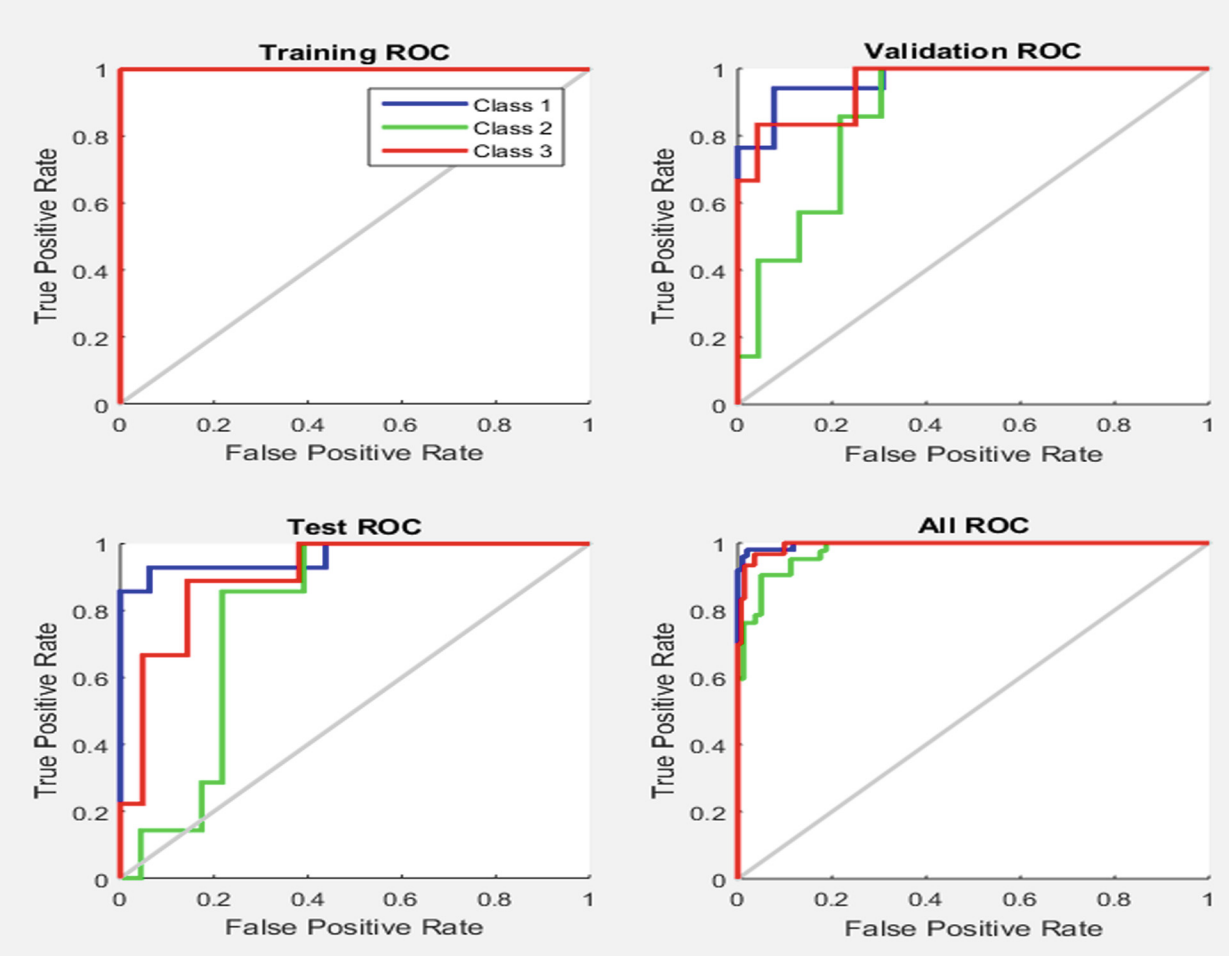
ROC results

The ROC curve (Fig. 10) is a graphical tool allowing presenting the capacity of a test to discriminate between different classes. It plays a huge role to depict TP rate against FP rate in medical statistics and more specifically in the field of ECG signals classification. Indeed, within our proposed ANN, we have an excellent rate of classification as seen in the ROC and a perfect prediction would yield an AUC of 0.93, presenting a value close to 1 for the training process, blue coloured in the curve. Similarly, test and validation process present high accuracy rate according to the curve.

#### Cross Entropy Results

**Fig. 11.**
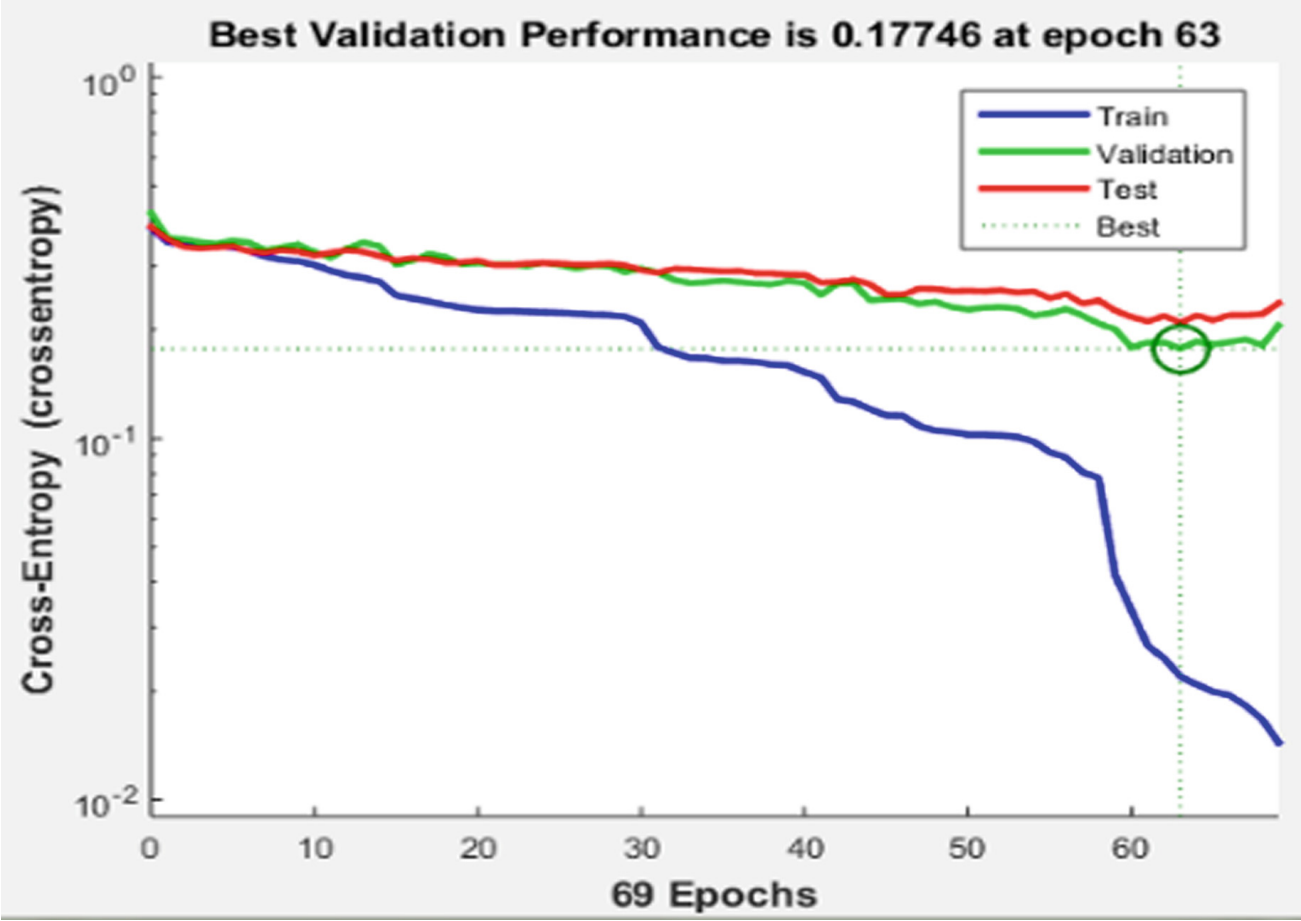
Best performance results

Table 3 shows the cross entropy and errors result through the training, the validation and the testing neural network process. Indeed, it is important to have low values of cross entropy to achieve good classification results. As presented in Fig. 11, the cross entropy value is roughly low which proves the efficiency of our model. Accordingly, Percent error indicates the fraction of samples, which are misclassified. A value of 0 means no misclassifications, 100 indicates maximum misclassifications.

**Table 3.** Cross-entropy and error results

Process	Numbers of records	Length of each record	Cross-entropy	Error
Training	142	10-s	5.91e^−1^	**0**
Validation	30	10-s	1.94e^0^	20
Testing	30	10-s	1.95e^0^	26.7

#### Processing Time

Experimental results obtained from using the MIT-BIH Arrhythmia Database showed that the processing time efficiency of our system can be highly increased by implementing the algorithm on GPU instead of the actual CPU. By using CPU, our algorithm took 2.052 s to make the real time classification of an ECG signal. Nevertheless, further improvement can be done on this method to achieve higher accuracy with less processing time.

## Discussions

The ANN application for ECG signals classification demonstrates greater accuracy compared to the state of the art, which proves that the ANN system is able to get more and more advanced results. Indeed, the state to have 100% of accuracy and 0% of error is hard to achieve. Despite this problem, ANN achieves high accuracy in comparison with related works. In [2], authors achieved 86% of accuracy. Accordingly, our ANN method overcomes results depicted in [2] with 7% of accuracy for ECG signals classifications. Indeed more, we are going deeper through layers, more accuracy results are better. We have achieved the top accuracy for MIT-BIH-ECG signal classification using the artificial neural network model by a value of 93.1% of accuracy where CNN comes with the third top accuracy with 92.7%. In Fact, we overcome [9], where authors proposed an Echo state neural network, with 92.7% of accuracy. Moreover, we surpass the state of the art in [11] with more than 3% of accuracy, and we have an error close to zero in histogram error detection with a low value of cross entropy, which proves the robustness of our classifier model. To conclude, the achieved results as depicted in Table 4 are comparable with the state of the art in fully automatic ECG classifiers and even outperform other ECG classifiers that follow more complex feature-selection approaches. Indeed, as presented in Table 4, we achieved encouraging results coming up to 93%, close to ECG –Net that is very complex and time consuming (it is using a huge amount of parameters and samples leading to increase the network complexity). Thus, there is no doubt to say that we succeed to better compromise between the testing accuracy and the network parameters complexity.

**Table 4.** Comparative study with the state of the art

Method	Best accuracy	Time process
Our work: **ANN1**	**93.1%**	**2.052** **s**
CNN + FCN layers [2]	86%	–
DenseNet [11]	89.5%	–
SVM [3]	87.5%	….
Echo state networks [9]	92.7%	…..
ECG-Net [13]	**94.0%**	……

## Conclusion

In this paper, we have achieved the top accuracy of MIT-BIH-ECG signal classification using the artificial neural network model by a value of 93.3% of test-accuracy, of sensitivity and of precision. Accordingly, the ROC shows an excellent curve of rate classification results. Accordingly, the histogram presents 0.07% error, which overcomes the state of the art without a huge number of feature extraction and without pre-processing stage. Our method is promising and can help clinicians to determine the class of each ECG patient. Indeed, for going faster, the next step will be dedicated to our application implementation on GPU and then on an FPGA.
